# Effects of Electroacupuncture Stimulation at “Zusanli” Acupoint on Hepatic NO Release and Blood Perfusion in Mice

**DOI:** 10.1155/2015/826805

**Published:** 2015-01-08

**Authors:** Shu-you Wang, Dong Zhang, Li-mei Tang, Shun-yue Li, Mei Wen, Xiao-jing Song

**Affiliations:** Department of Biomedical Engineering, Institute of Acupuncture and Moxibustion, China Academy of Chinese Medical Sciences, Beijing 100700, China

## Abstract

The study is to observe the influence of electroacupuncture (EA) stimulation at “Zusanli” (ST36) on the release of nitric oxide (NO) and blood perfusion (BP) in the liver and further explore whether the hepatic blood perfusion (HBP) changes were regulated by EA ST36 induced NO in nitric oxide synthase inhibited mice. The HBP change of the mice was detected by laser speckle perfusion imaging (LSPI) before and after being given interventions, and the NO in liver tissue was detected by nitric acid reductase in each group. The NO levels and HBP in the L-NAME group were significantly lower than those in the control group (*P* < 0.01). The NO level and HBP increase in EA group were significantly higher than those in control group (*P* < 0.05). The NO level in the L-NAME EA group was slightly higher than that in the L-NAME group. The HBP increase in the L-NAME EA group was not statistically significant. These results showed that EA could accelerate the synthesis of NO and thereby increase HBP via vasodilation in liver tissue.

## 1. Introduction

The liver is an organ that is very rich in blood in the circulation of the body and has unique circulation physiological characteristics [1]. A lot of studies have shown that the physiological function and pathological change of the liver are closely related to the blood circulation function of the body [2]. In the liver diseases, the mechanisms of the liver tissue damage are mainly related to the factors such as microcirculation dysfunction, overproduction of oxygen radicals, exhaustion of energy substance, calcium overload, and mitochondrial dysfunction. Among these factors, the microcirculation dysfunction plays an important role and largely decides the damage degree of the liver tissue [3]. Therefore, hepatic microcirculation is a very significant biological indicator in monitoring the liver disease progress and treatment processes.

Many factors and bioactive substances participate in regulating the physiological and pathological processes of the microcirculation system of the body [4, 5]. As an active signaling and effector molecule in the organism, nitric oxide (NO) plays an important role in the maintenance and regulation of the microcirculation function of the visceral organ [6–9]. The vascular endothelial cells are the primary cells to synthesize NO in the body. NO synthesized by vascular endothelial cells wields a significant influence in regulating vasomotion and maintaining blood coagulation-thrombolysis balance. Hyposecretion or inactivation of NO which is induced by vascular endothelial dysfunction is regarded as the important virulence factor involved in the pathogenesis and progression of vascular diseases [10, 11]. Activating the endothelial nitric oxide synthase (eNOS) in the vascular endothelial cells will promote the synthesis and release of NO which can penetrate the cell membrane and diffuse to the adjacent smooth muscle cells. In the smooth muscle cells, NO binds to the Fe^2+^ which is the hemoglobin component of the guanylate cyclase followed by elevating the cGMP levels, activating the protein kinase, phosphodiesteric acid, and ion channels, resulting in the vessel smooth muscle relaxation and vasodilatation, and increasing blood perfusion (BP) [12–15].

The studies have shown that acupuncture has obvious effects on improving microcirculation and increasing the BP of the visceral organ and local tissue [16], and our laboratory has done a lot of work on this field [17–19]. The existing researches have shown that the NO level of tissues or organs could be regulated by acupuncture [20, 21]. However, related studies are absent on which link in the acupuncture will motivate the regulation of the synthesis and release of NO. In this study, we try to observe the influence of electroacupuncture (EA) on the NO concentration and BP in the liver tissue of the mice through inhibiting the activity of eNOS and discuss the relationships between NO levels and hepatic blood perfusion (HBP) regulation by EA.

## 2. Materials and Methods

### 2.1. Animal Preparation

Forty healthy male Kunming mice weighing 20–22 g, average age of 3 months, were randomly allocated into EA, L-NAME, L-NAME EA, and control groups, 10 mice in each group. The animals were provided by the Animal Center of Chinese Academy of Medical Sciences. The animals were fed as experimental animals and treated according to the accepted international criteria. The experimental procedure should be conducted according to the Animal Ethics Committee of Chinese Academy of Medical Sciences.

The animals were anesthetized by intraperitoneal injection of 2% pentobarbital sodium (45 mg/kg^−1^, Sigma-Aldrich, St. Louis, USA). Then the mice were injected intraperitoneally (i.p.) with physiological saline (15 mg kg^−1^) in EA and control groups. The mice were injected intraperitoneally (i.p.) with 0.06% N^G^-nitro-L-arginine methyl ester, hydrochloride (L-NAME) (15 mg kg^−1^, Sigma Company, USA) in L-NAME and L-NAME EA groups. In addition, EA was given at bilateral Zusanli (ST36) after injection 40 min in EA and L-NAME EA groups' mice.

### 2.2. EA Stimulation

For the EA and L-NAME EA groups, bilateral ST36 which was located at the posterolateral knee of hind limbs, about 2 mm below the fibular head, was inserted with number 32 needle (0.18 × 13 mm, Suzhou, China) to 3 mm depth. Then, the needles were connected to the EA device (MBT-1 device for microcomputerized pulse-synchronized treatment, Zhejiang Huayin Electronic Co., Ltd., China). The stimulation time was 30 min, the current intensity was 4–6 V, and the pulse frequency was 2 Hz.

### 2.3. HBP Measurement

Under anesthesia, the mouse was fixed on a plank in a supine position. Approximately 1 cm of lineal incision below the xiphoid process and along the ventral median line was made. Then the liver tissue was exposed. The underneath liver lobe was separated and plainly placed on the top of a bracket above the abdomen. Then the mouse model was placed in the experimental constant temperature box whose temperature was kept at 30–32°C and humidity was 80–90%. The liver lobe was placed about 28–30 cm below the laser scanner. The box was placed in a shielding chamber without sunshine, infrared radiation, and ventilation.

Moor-FLPI laser speckle perfusion (LSP) imager (Moor instruments Ltd., Axminster, UK) was used in this study. The scanning model was of low density and 25 fps, the time interval was 1 s, exposure time was 20 ms, and 10 frames were continually scanned at each time point (10 frames were averagely processed into a single frame to obtain the mean HBP at each time point). For the control and the L-NAME group, the HBP images were recorded before and after investigation 30 min. For the EA and L-NAME EA groups, the HBP images were recorded before and after EA 30 min. Then the HBP images were saved and analyzed by the Image Review Program of Moor-FLPI-V2.0 software. The location, range, and degree of the HBP of the three groups were compared at each time point. The round region of interest (ROI) with the same area in each LSP image was selected for measuring the HBP.

### 2.4. NO Assessment

After the HBP detection was finished for all the groups, the scanned lobe of liver (about 0.5 g) was taken, homogenized with adequate 0.89% cold saline, and centrifuged. Nitrate reductase method and type 7160 spectrophotometer (Hitachi, Japan) were used to determine the NO content of the liver tissue of mice (NO reagent kit, Nanjing Jiancheng Bioengineering Institute, China).

### 2.5. Experimental Flow Chart

See Figure 1.

### 2.6. Statistical Analysis

The mean HBP was, respectively, calculated with perfusion unit (PU for short) as the unit. The difference of HBP before and after 30 min and the level of NO in each group were expressed as mean ± standard deviation. Then the SPSS17.0 software was used for paired *t*-test of the same index between groups. *P* < 0.05 was regarded as significant difference.

## 3. Results

### 3.1. Comparison of NO Contents in Each Group

**Figure 2:**
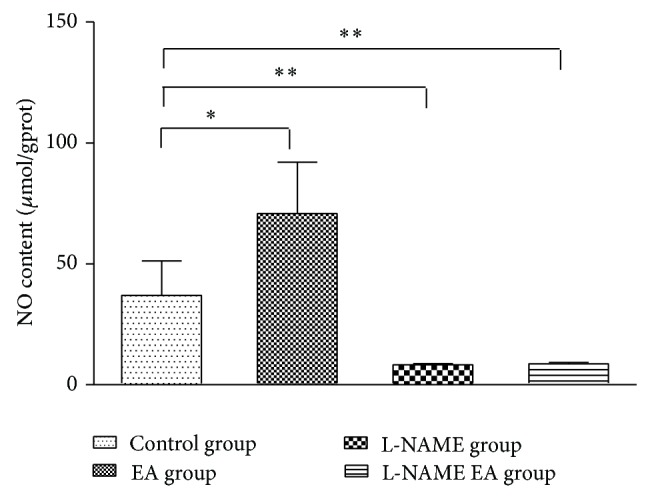
Comparison the NO contents of liver tissues of mice among four groups.* Note.* ^*^ *P* < 0.05; ^**^ *P* < 0.01.

Data describing the levels of NO from four groups were summarized in Figure 2. The NO content of liver tissue in the EA group (70.76 ± 21.33 *μ*mol/gprot) was significantly higher than that in the control group (37.01 ± 14.23 *μ*mol/gprot) (*P* < 0.05). After injection of L-NAME, the NO content in the L-NAME group was 8.27 ± 0.43 *μ*mol/gprot. It was significantly lower than that in the control group (*P* < 0.01). The NO content in the L-NAME EA group (8.65 ± 0.56 *μ*mol/gprot) was slightly higher than that in the L-NAME group, but there was no statistical difference between the two groups (Figure 2).

### 3.2. Analyses of LSP Images of the Liver

**Figure 3:**
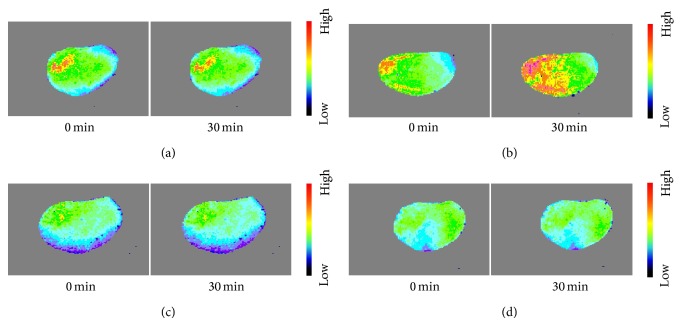
Hepatic LSP images of mice in four groups.* Note*. The liver LSP images of mice in control group (a), EA group (b), L-NAME group (c), and L-NAME plus EA group (d) at 0 min and 30 min, respectively. The color scale from the cold to the warm indicates the blood perfusion ranging from the low to the high level. In the control group, LSP image appeared largely as light green with small amount of yellow and red. Generally, the color distribution remains unchanged. In the EA group, the red region became enlarged immediately after 30 min of EA stimulation. In both L-NAME group and L-NAME plus EA group, the majority region of LSP images was light green, with slight change of the color distribution between acquisitions at 0 min and 30 min time points.

The cold color indicates that the BP is low, and the warm color indicates that the BP is high in the LSP images. From the LSP image before and after 30 min of investigation, it could be seen that the colors were distributed evenly in control group, indicating that the HBP changes were small during 30 min (Figure 3(a)). In the EA group, the orange and red regions appeared increasing significantly after EA 30 min, thus indicating that the HBP was significantly increased after EA (Figure 3(b)). In the L-NAME group, the changes of LSP images were similar to the control group before and after 30 min, indicating that the level of HBP was stable after intraperitoneal injection of L-NAME (Figure 3(c)). In the L-NAME EA group, the cold tonal regions of the LSP image decreased slightly after EA 30 min, indicating that the HBP increases were just small (Figure 3(d)).

### 3.3. Quantitative Analyses of HBP Changes

**Figure 4:**
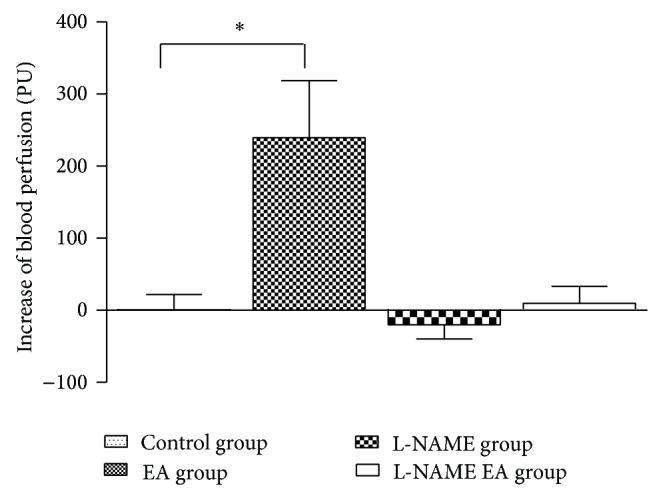
Comparison of HBP differences of mice among four groups.* Note*. ^*^ *P* < 0.05.

The data describing of HBP has shown that, except in the L-NAME group, the HBP in the control group, EA group, and L-NAME EA group all increased after observation of 30 min. In the L-NAME group, the HBP reduced slightly (−20.05 ± 19.60 PU) after 30 min injection of L-NAME. The highest increase was found in the EA group, which was 239.32 ± 79.26 PU. The increase of HBP was only slightly in the L-NAME EA group (9.80 ± 23.37 PU). The HBP barely changed in the control group (0.81 ± 20.87 PU). There was significantly statistical difference of BP increase between the EA group and control group (*P* < 0.05). But there was no statistically significant difference of BP increase between the L-NAME group and control group and also between the L-NAME group and L-NAME EA group (Figure 4).

## 4. Discussion

The previous studies showed that acupuncture stimulation may activate multisystem reactions of the body. The regulatory effect of acupuncture on the vascular function is well recognized in recent studies. Either moxibustion, EA, or acupuncture stimulation can increase the blood flow in the local and distant skin, muscles, and even visceral organs and significantly increase the blood flow velocity, improve the blood flow morphology, and increase the number of the opened peripheral capillaries and so forth [22–25]. The renormalizing of the damaged and pathological cells was promoted through the improvement of the circulatory system function and enhancement of the metabolism of the tissue and organ. The liver is an important organ which has profuse blood flow. The hepatic microcirculation is a very significant biological indicator in the diagnosis and treatment of liver disease. HBP is an important parameter in the diagnosis of the portal hypertension and evaluation of operative efficacy of cirrhotic portal hypertension and hypersplenism [26–28]. The study of the regulating effect of acupuncture stimulation on the HBP plays an important role in applications of the acupuncture therapy in prevention and treatment of liver diseases.

The traditional meridian theory indicates that the circulations of stomach meridian and liver meridian are both in contact with stomach. The Qi and Xue of the two meridians can run through stomach and communicate with each other. Therefore, the functions of organs belonging to liver meridian can be regulated by acupuncture stimulation ST36. Clinically, ST36 is an important point used to treat liver disease in traditional Chinese medicine therapy [29]. Some studies reported that EA can increase the BP of gastrointestinal and liver surface [16]. We hypothesize that NO as a vasodilator substance with stronger activity would be associated with changes of BP by EA ST36. So, the influence of EA ST36 on the NO level of liver and HBP was observed in this experiment.

NO which is produced through the catalytic decomposition of L-arginine by NOS is a vasodilator with stronger activity in the living body. Changes of the activity of NOS are a key enzyme for synthesis of NO directly affecting the NO level. At present NOS has at least three known subtypes: endothelial nitric oxide synthase (eNOS) which is mainly expressed in the vascular endothelial cells [30]; neuronal nitric oxide synthase (nNOS) which is mainly expressed in brain and nervous tissues; and inducible nitric oxide synthase (iNOS) which is mainly expressed in the macrophage and does not exist in the normal cells. Usually, there are only eNOS and iNOS in the liver tissues. The synthesis of NO catalyzed by eNOS can dilate the blood vessel and maintain the normal microcirculation of the liver [31]. It was found that the NOS expression was increased where the hepatocellular damage was the most serious around the ischemic and anoxic central veins in concanamycin-induced liver injury. The high level of NO dilated the blood vessel to accelerate blood flow rate and alleviate ischemic and anoxic hepatocyte [32]. Previous studies indicated that acupuncture stimulation of the ST36 could enhance the expression of NOS and elevate the levels of NO in the skin points, central nervous system, or the peripheral blood [33, 34]. It had been observed in this experiment that EA ST36 could significantly elevate the NO level of mice, coupled with the HBP increase. NO mediates the relaxation of vascular smooth muscles and vasodilation as a second messenger. The blood perfusion of the tissue is closely related to the NO concentration [35]. With these data, we considered that EA ST36 had activated the various sensory receptors in the acupoint area; then the acupuncture signal was transmitted into the liver to activate the vascular endothelium of the liver tissue. The eNOS expression in the endothelial cells was boosted; at the same time the synthesis and release of NO were increased. Under physiological conditions, a certain NO level in the tissues or organs can promote the blood circulation. High concentration of NO could promote the relaxation of the vascular smooth muscle and finally lead to the hepatic vascular dilation and HBP increase.

It also had been observed that, after intraperitoneal injection with L-NAME, the levels of both NO and the HBP decreased. L-NAME is a nonselective NOS inhibitor which mainly produces the inhibitory effect on eNOS. The synthesis of NO could be effectively inhibited by L-NAME in the body. The studies have shown that L-NAME can inhibit the activity of mitochondrial NOS after acute lung injury and reduce the formation of NO to protect the integrity of the morphology and function of mitochondria [36, 37]. Local inhibition of nitric oxide attenuates cutaneous vasodilator responses during postmenopausal hot flashes [38]. Otherwise, skin temperature, core body temperature, heart rate (HR), and blood pressure during warm moxibustion-like thermal stimulation at the site perfused with L-NAME were significantly decreased in healthy people [39]. These reports demonstrate that NO which is involved in the mechanism of visceral or local tissue vasodilation can be inhibited by L-NAME. After intraperitoneal injection with L-NAME, the activity of NOS in the mice, especially the activity of eNOS, was so strongly inhibited that it lost the ability to catalyze the synthesis of NO. The NO level in the liver tissues reduced. So its function that regulated vasodilatation was weakened. Finally, the HBP correspondingly decreased due to vasodilation suppression induced by the low level of NO in liver tissues of L-NAME group's mice. When EA stimulation was applied after injection with L-NAME, the liver NO content and HBP of the mice were still lower than those of EA group. After intraperitoneal injection with L-NAME, the eNOS activity that had been strongly inhibited in the body could not be motivated by EA stimulation. Therefore, the generation of NO in liver tissues could not be enhanced by EA. There was no significant change of the HBP in the L-NAME EA group.

The synthesis of the NO, the maintenance of NO concentration, and vascular regulation in the body tissue are very complex biological processes. More studies which are related to the mechanisms of the effect of EA on NO and vascular activity need to be conducted in future work.

## 5. Conclusion

The NO content in the liver tissues and the HBP can be increased by EA ST36 in mice. In contrast, L-NAME inhibition can lead to suppression or disappearance of the effects of EA ST36. Therefore, HBP increased after EA ST36 possibly by accelerating the synthesis of NO to mediate vasodilation in liver tissue of mice. This result has provided new experimental information for the mechanism of hepatic circulation by EA.

## Figures and Tables

**Figure 1:**
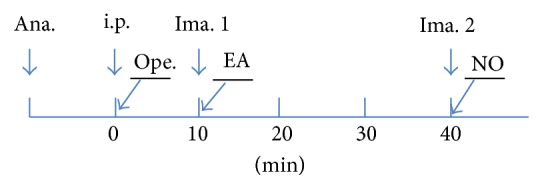
Flow chart of the experiment.* Note*. Ana.: anesthesia; i.p.: intraperitoneal injection; Ope.: liver model operation; Ima. 1: the first HBP image acquisition; EA: EA stimulation onset; Ima. 2: the second HBP image acquisition; NO: NO measurement.
